# Effect of topical local anaesthesia on injection pain associated with administration of sterile water injections - a randomized controlled trial

**DOI:** 10.1186/s12871-022-01573-0

**Published:** 2022-02-01

**Authors:** Lena B. Mårtensson, Britt-Marie Gunnarsson, Sandra Karlsson, Nigel Lee, Ingrid Bergh

**Affiliations:** 1 School of Health Sciences, University of Skövde, P.O. Box 408, SE-541 28 Skövde, Sweden; 2 School of Nursing, Midwifery and Social Work University of Queensland, Chamberlain Building, University of Queensland,, St Lucia, Queensland 4072 Australia; 3 School of Health and Welfare, Jönköping University, P.O. Box 1026, SE-551 11 Jönköping, Sweden

**Keywords:** Pain, Pain relief, Sterile water injections, Randomized controlled trial, Childbirth

## Abstract

**Background:**

Sterile water injections can provide effective pain relief during childbirth, particularly for low back pain related to childbirth. However, the pain associated administering the injections can negatively impact women’s impressions of the procedure. It may discourage women from considering repeat doses despite the quality of analgesia experienced. Determining strategies to reduce the pain related to the administration of sterile water injections would improve the acceptability of the technique. Therefore, the aim of this study was to evaluate the effect of topical local anesthesia on the pain associated with administration of sterile water injections.

**Methods:**

The study was designed as a multi-arm single-blind, randomized, controlled trial and 120 female healthy students were randomly divided according to one of four groups. The Intervention group received sterile water injections with topical local anesthesia. Control group 1 received sterile water injections without topical local anesthesia, control group 2 received injections of isotonic saline 0.9% with topical local anesthesia and control group 3 received injections of isotonic saline 0.9% without topical local anesthesia. Pain Immediately after the injections and subsidence in pain were recorded using a visual analogue scale. Sensations in the injection area were reported 15 min and the day after the injections.

**Results:**

The main finding of this study was that local anesthesia with EMLA® reduces the pain associated with the administration of intracutaneous sterile water injections. There was a significant difference in the self-assessed pain score immediately following the injections between the control (73.3 mm) and intervention groups (50.0 mm), *p* = 0.001. No adverse side effects were reported.

**Conclusion:**

Local anesthesia with EMLA® reduces the pain associated with intracutaneous administration of sterile water injections.

**Trial registration:**

The study was registered 08/07/2014 at ClinicalTrials.gov Identifier: NCT02213185.

## Background

Most women in childbirth experience intense pain. This kind of pain can affect the women’s experience of childbirth negatively for up to 1 year postnatally [1]. The pain may even result in symptoms of post-traumatic stress [2]. Safe and effective pain relief should therefore be available for all women. However, many women are reluctant or hesitant to accept pharmacological pain relief out of concern for possible adverse side effects on themselves or their unborn baby [3]. Since the mid-1980s, sterile water injections (SWI) have been used to alleviate low back pain during childbirth [4]. In addition to treatment for childbirth pain, SWI has been used to relieve chronic neck pain, renal colic [5] and ureterolithiasis during pregnancy [6].

Along with several smaller studies, a large double-blind, randomized controlled trial successfully demonstrated that SWI could provide effectively relief for low back pain during childbirth [7]. Clinical guidelines for the use of the procedure have earlier been published [4]. Previously it has been reported that the pain associated with administering the injections can negatively impact women’s experiences [8, 9]. It has also been reported that women rated the injection pain as more painful than labor contractions [10]. Further, midwives have reported reluctance to recommend this treatment due to the pain inflicted during the administration of the injections [11].

**Fig. 1:**
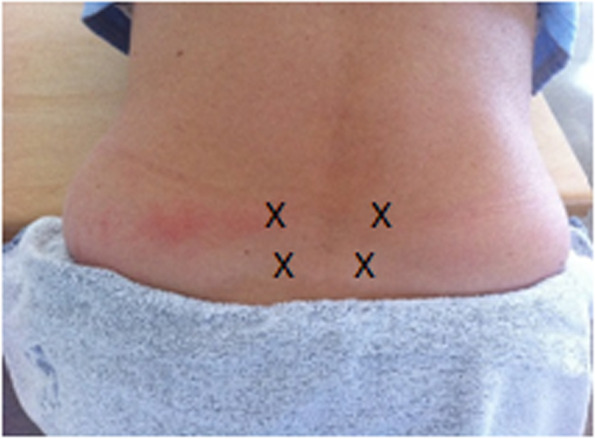
Locations of the injections

SWI is a simple and inexpensive method involving the injection of small volumes of sterile water intracutaneously (0.1 ml) or subcutaneously (0.5 ml) at the site/s at which the woman perceives pain, Fig. 1. The most common technique entails the administration of four injections, but three studies demonstrated a good effect after just one injection [12–14]. Whether pain relief is influenced by the number of injections or the volume of sterile water per injection has been discussed [15, 16]. However, Lee et al. [14] found that four injections resulted in better pain relief effect than one injection. Pain relief usually occurs within a few minutes and can be repeated if needed [17, 18]. The only observed disadvantage of SWI is the injection pain. The pain can be described as similar to a bee or wasp sting, with a duration of approximately 20–30 s [4]. In an earlier cross-over study including 100 non-pregnant healthy women reported that the injection pain was considerably reduced if the injections were administered subcutaneously using a somewhat more substantial volume of sterile water [19].

Moreover, subcutaneously SWI in laboring women seems to give a similar pain relief effect compared to intracutaneous injections [20]. However, in clinical practice, women still find subcutaneous injections painful, especially when receiving multiple injections. It is therefore relevant to question if local anesthetic can reduce the pain related to the injections. Byrn et al. [21] advised against administering local anesthetic before SWI but provided no evidence or physiological basis to support this recommendation. In a later study, Iwama et al. [22] showed excellent relief from myofascial pain when the local anesthetic was combined with sterile water or isotonic saline. However, the trial did not include a comparative non-anesthetic control group. The anti-nociceptive mechanisms of SWI are not fully understood, but some theories are found in the literature. One is the gate control theory [23], another is descending pain relief system [24] and finally, the diffuse noxious inhibitory control (DNIC) [25]. Moreover, not much is known about how fluid is absorbed in the tissue during intradermal injections, and there are no known in depth-studies showing flux characteristics in the tissue [26].

The pain associated with administering water injections is now arguably the main deterrent to more widespread acceptability and use of this method. A small number of studies suggest the use of local anesthetics may be of use in this regard. However, the inclusion of injectable drugs, such as lidocaine, also involves some injection discomfort. Furthermore, no studies have yet tested this approach against a placebo control. The use of topical anesthetics may avoid the inherent injection pain of local anesthetics providing a suitable alternative. Therefore, this study aimed to evaluate the effect of topical local anesthesia on pain associated with the administration of sterile water injections.

## Methods

### Aim

The aim of this study was to evaluate the effect of topical local anesthesia on the pain associated with the administration of sterile water injections.

### Study design

The study was designed as a single-blind, randomized, controlled trial with four arms following the CONSORT guidelines [27]. Data collection took place from September 2014 to December 2019.

### Participants and recruitment

As there is some evidence to suggest a gender difference in the perception of pain, female participants were used to more closely reflect the possible effect of the intervention in laboring women [28]. A previous study with a similar design [19] also showed that it is possible to recruit students even if the trial could involve an experience of pain during the administration of injections. Recruitment of voluntary female students took place at one University in the Western part of Sweden.

Information about the study was first given on the student noticeboards. However, this approach was not practical, and only a small number of students responded. Therefore, we changed to more targeted information. Nursing and midwifery students received information about the study during an appropriate course introduction during their first week at the University. Interested students were requested to put their names on a list. They were also informed that the registration was not binding. The students were then contacted, and if they still were interested in participating, a time for scheduling the injections was decided. It was not possible to know how many of the nursing and midwifery students fulfilled inclusion criteria. Therefore, it is impossible to identify how many students were eligible to participate in the study. Oral and written information was once again given just before the injections were given. A further check was made to ensure that all inclusion criteria were met. The exclusion criteria were based on conditions that possibly could have an impact on the experience of pain [29, 30].

### Criteria for inclusion

- Female
- Age 18–45 years
- Substantially healthy (self-reported)
- Sufficient knowledge of the Swedish language to understand written and oral instructions.

### Criteria for exclusion

- Pregnancy
- Hypersensitivity to Lidocaine and Prilocaine
- Previous experience of SWI
- Ongoing pain
- Use of medication for depression, pain, or sleeping disorder 24 h before the experiment
- Smoking, snuffing (a type of tobacco that is kept in the mouth), physical activity, and intake of caffeinated beverages (coffee, tea, or energy drink (e.g., Red Bull)) 2 h before the experiment.

### Randomization and procedure

A randomization protocol was created by independent statisticians using random number generator software. Information about the treatment group, to which the women had been randomized, was kept in prepared non-transparent envelopes containing study protocol and questionnaire.

### Primary outcome

- Pain immediately after administration of the injections.

### Secondary outcomes

- Subsidence in pain
- Sensations in the injection area were reported 15 min after injections
- Sensations in the injection area were reported the day after injections

For many years, topical preparations such as EMLA® have been routinely used in Swedish hospitals as a local anesthetic to relieve the pain associated with various forms of injections. An EMLA® patch consists of a mixture of 25 mg lidocaine and 25 mg prilocaine. EMLA® usually provides appropriate analgesia within 1 to 2 h, and the effect lasts up to 2 h after removal of the patch [31]. An EMLA® patch provides skin analgesia corresponding to a depth of 2.9 mm after 60 min of application and 4.5 mm after 120 min [32], which means that, in this particular situation, intracutaneous injection was preferable to subcutaneous. A patch without anesthetics was used as placebo in control groups 2 and 3. Isotonic saline (NaCl 0.9%) was used since the percentage of salt in isotonic saline is similar to in the body. The latter means that the isotonic saline does not cause the same degree of osmotic irritation as sterile water, which is salt-free [19]. However, the pain related to the needling of the skin is the same irrespective of the fluid thereafter injected. After providing informed consent, participants meeting the inclusion criteria was randomized into one of four groups:

### Intervention group (EMLA SWI)

Four EMLA® patches were applied to the skin of the lower back (Michaelis Rhomboid; Fig. 1). After 1.5 h, the patches were removed, and one injection of sterile water (0.1 ml) was given intracutaneously at each of the patch locations, in a total of four injections.

### Control group 1 (placebo SWI)

Four placebo patches were applied to the skin of the lower back (Michaelis Rhomboid; Fig. 1). After 1.5 h, the patches were removed, and one injection of sterile water (0.1 ml) was given intracutaneously at each of the patch locations, in a total of four injections.

### Control group 2 (EMLA NaCl 0.9%)

Four EMLA® patches were applied to the skin of the lower back (Michaelis Rhomboid; Fig. 1). After 1.5 h, the patches were removed, and one injection of isotonic saline (NaCl, 0.9%) (0.1 ml) was given intracutaneously at each of the patch locations, in a total of four injections.

### Control group 3 (placebo NaCl 0.9%)

Four placebo patches were applied to the skin of the lower back (Michaelis Rhomboid; Fig. 1). After 1.5 h, the patches were removed, and one injection of isotonic saline (NaCl, 0.9%) (0.1 ml) was given intracutaneously at each of the patch locations, in a total of four injections.

### Instruments

#### Visual analogue scale

A horizontal Visual Analogue Scale (VAS) was used to assess the primary outcome, the perceived pain related to the injections. VAS is a 100-mm-long, ungraded vertical or horizontal line with the suggested endpoints ‘*no pain*’ (left) and ‘*worst imaginable pain*’ (right) [33]. The VAS is sensitive to pain intensity [34–36], and most individuals have no difficulties using it [35, 37]. VAS scores can also be divided into three main categories. i.e. mild pain (< 30 mm), moderate pain (31-70 mm) and severe pain (> 70 mm) [38]. The participant marked an appropriate point on the horizontal scale with a vertical line.

#### Case report form

The study protocol consisted of ten pages, one for each time-point of the measurements point (immediately after the injections, 30 s after the injections, and then after 1, 2, 3, 4, 5, 10 and 15 min). The participants could not see their previous care which might influence the current score. In the end of the form there was an open-ended question regarding experienced sensation in the injection area.

#### Questionnaire

The questionnaire contained questions about age, parity, marital status, education, other occupation, menstrual cycle patterns and date of last menstrual period.

### Data collection

All participants were registered by date, name and date of birth in a logbook. The code on the envelope, study protocol and questionnaire were also registered. No names or date of birth was recorded in the study protocols or questionnaires, only the code, which makes it possible to identify the participants in the logbook. At the time of allocation, the person responsible for data collection selected the envelope with the lowest number. In the envelope there was information about which patches and injections the participant were to receive. The participants were unaware of the group they were randomized to. The logbook was kept in a locked cupboard, separate from study protocols and questionnaires.

After randomization, the participants scored any current pain they were experiencing on a VAS to exclude those with pre-existing or ‘ongoing pain’. After that, four EMLA® patches were applied to the skin of the low back (Michaelis Rhomboid, Fig. 1) in the intervention group and the control group 2. In the control group, 1 and 3, four placebo patches were applied in the same way. The women were then requested to complete the questionnaire. After 1.5 h, the patches were removed, and one injection of 0.1 ml sterile water (in the intervention group and the control group 1) or 0.1 ml NaCl, 0.9% (in the control group 2 and 3) were given. All injections were given intracutaneously at each location where the patches were placed. In total, four injections were given to all women. Two of the researchers administering two injections each at the same time. All injections were given via a 1 ml Mantoux plastic syringe and a thin needle (B. Braun Omnifix; diameter: 0.40 mm, length: 20 mm). Immediately after the injections had been given (approx. 1–3 s), the participants were asked to score the pain related to the injections on a VAS. The perceived pain in the injection area was repeated 30 s after the injections, and then after 1, 2, 3, 4, 5, 10 and 15 min. Fifteen minutes after the injections were given, the women were asked to describe, in their own words, if they experienced any sensations, at that time point, in the injection area. All participants received an email 1 day after the procedure in which they were asked to describe if they, at that time point, had any sensations in the injection area.

### Ethical issues

SWI could be experienced as painful by the women in the group not treated with EMLA®. It was assumed that women with an extreme fear of needles and injections are unlikely to volunteer and so will be self-excluded from the study. Accurate information about the protocols was given to those who accepted the invitation to participate. No side effects, except for pain during injections, have earlier been reported in clinical practice. The small risk of infection was minimized via a good aseptic technique. The reason to first conduct this study with volunteers rather than women in childbirth was important. If local anesthesia with EMLA® is found to reduce the injection pain associated with SWI, the next step would be to investigate whether this technique also gives adequate relief of low back pain during childbirth. The participants in the present study did not receive a financial incentive. The Central Ethical Review Board in Sweden approved the study (Dnr Ö 9–2013).

### Analysis

#### Sample size

Mårtensson and Wallin [20] found that female students receiving intracutaneous sterile water injections reported an average pain score of 61 (mm) (SD = 19) on a 100-mm VAS. Based on that study, it was estimated that the present study would require 18 subjects in each group to achieve 90% power, at a two-sided 0.001 significance level, to detect a decrease in pain of 32 mm (VAS). This reduction in pain may be relevant since the pain would be experienced in the range of mild to moderate pain (VAS) [39]. Including 30 patients per group (a total of 120) compensated for dropout.

### Statistical analysis

The VAS score was measured in millimeters from the left anchor marked ‘no pain’ and the point scored by the participant [40]. Differences between the groups in demographics were tested using Fisher’s Exact Test (dichotomous variables) (two and four groups), Mann-Whitney’s test (two groups) and Kruskal–Wallis H-test (four groups) (categorical data) and t-test (two groups) and one-way ANOVA (four groups) (continuous variables). Mann-Whitney’s U-test was also used to compare perceived pain (VAS scores), and within the injection area over time, between the EMLA-SWI and placebo SWI groups and between EMLA-NaCl and placebo NaCl group. To test differences between proportions Z-test for 2 independent proportions was also used. VAS scores are also presented as mean and standard deviation. Since the aim of this study was to evaluate the effect of topical local anesthesia on the pain associated with administration of sterile water injections, no comparisons were made between the SWI and NaCl groups. In keeping with the sample size calculation, a two-tailed test at the 0.001 significance level was used.

## Results

**Fig. 2:**
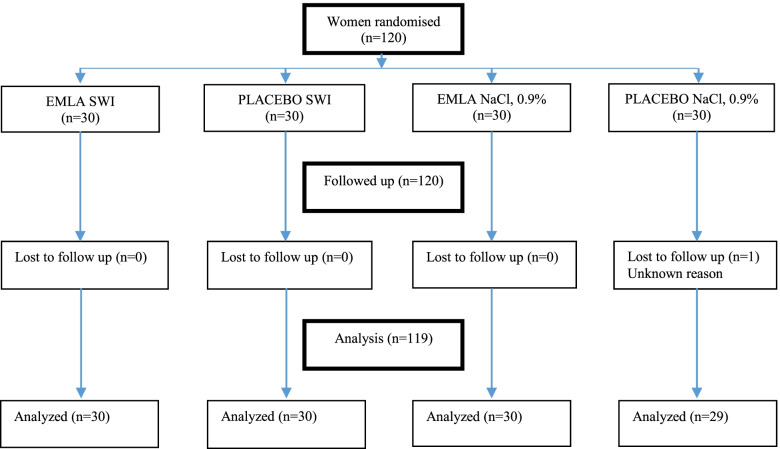
Flow chart of the study. SWI=Sterile Water Injections, NaCl, 0.9% = Isotonic saline. EMLA SWI=Intervention group, EMLA NaCl = Control Group 1, Placebo SWI=Control Group 2, Placebo NaCl = Control Group 3

A total of 120 female students participated in the study. For unknown reasons, one woman discontinued the participation. In total, 119 women were included in the analysis. The recruitment and flow chart of the study is presented in Fig. 2. There were no differences between the SWI groups or NaCl groups, nor between all four groups regarding socio-demographic variables (age; body mass index; knowledge of SWI; previous childbirth; anxiety for pain and use of tobacco), Table 1.

**Table 1:** Participant socio-demographic variables

	Intervention group EMLA SWI (*n* = 30)	Control group 1 Placebo SWI (*n* = 30)	Control group 2 EMLA NaCl (*n* = 30)	Control group 3 Placebo NaCl (*n* = 30)
*Age (years)*				
Mean	27,53	27,30	26,70	24,70
+ SD	6,01	6,88	6,48	4,38
*Body mass index*				
Mean	25,06	23,94	23,50	24,47
+ SD	4,21	5,87	2,61	3,89
*Knowledge of SWI*				
Yes	14	12	14	11
No	16	18	16	18
*Previous childbirth*				
Yes	10	9	7	7
No	20	21	22	23
*Anxiety for pain*				
Not worried at all	8	8	12	7
Not particularly	20	20	16	19
worried	2	1	2	3
Pretty worried	0	1	0	0
Very worried				
*Use of tobacco*				
Yes	4	9	5	7
No	26	21	24	23

*EMLA* Patch with local anesthesia, *SWI* Sterile water injections, *NaCl* isotonic saline (0.9%)

**Fig. 3:**
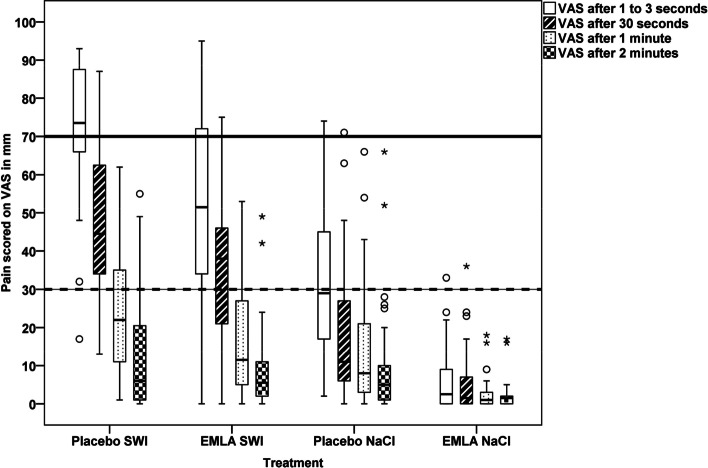
Distribution of Visual Analogue Scale (VAS) scores (millimeters (mm)) at the first four time-points; immediately after injection (1–3 s), 30 s, 1 min and 2 min. Note, in this figure the groups are sorted after pain intensity at injection time. The cut-offs for mild (< 30 mm), moderate (31-70 mm), and severe pain (> 70 mm) is market with reference lines (y-axis)

There was a statistically significant difference regarding perceived pain of 23.3 mm on VAS, 1–3 s after the injections of sterile water were given, between the intervention group (EMLA SWI) and control group 1 (placebo SWI) (50.0 vs 73.3, *p* = 0.001). This statistically significant difference does not remain 30 s after the injections. However, in both groups, the perceived pain has decreased, during the time of observation, Fig. 3 and Table 2. This trend was noted in the intervention group (EMLA and SWI) but did not achieve a statistically significant difference compared to control group 1 (placebo and SWI). Further, immediately after the injections were given (1–3 s), 30% (*n* = 9) scored severe pain in the EMLA SWI group. The corresponding figure for the placebo SWI group was 63.3% (*n* = 19), (*P* = 0.00634).

**Table 2:** Comparison between the PLACEBO and EMLA SWI groups. VAS scores (mm) presented as mean and standard deviation (+SD) at each time-point

	PLACEBO SWI	EMLA SWI	
Time-points	Mean (+SD)	Mean (+SD)	*p*-value
1–3 s	73.3 (18,9)	50.0 (26,7)	0.001
30 s	49.5 (20,8)	38.1 (20,0)	0.052
1 min	25.3 (18,0)	17.1 (15,3)	0.056
2 min	14.0 (16,3)	8,8 (11,8)	0.381
3 min	8.0 (12,6)	3.2 (5,9)	0.142
4 min	4.7 (6,1)	1.3 (2,0)	0.016
5 min	6.5 (9,0)	1.7 (3,5)	0.003
10 min	4.0 (5,6)	1.8 (4,7)	0.008
15 min	3.14 (6,1)	1.0 (2,1)	0.015

*EMLA* Patch with local anesthesia, *SWI* Sterile water injections

After 30 s, 6.7% (*n* = 2) in the EMLA SWI group and 20.7% (*n* = 6) in the placebo SWI group recorded VAS equal to severe pain, (*p* = 0.11642). At 1 min all women, in these groups, scored their pain as mild or moderate (Fig. 3).

While at the 1-min time point, none of the women across the four groups rated their pain as severe, the range of the VAS scorings indicates a considerable individual variation in how the women perceived their pain. This was especially pronounced in the EMLA SWI group at the first time-point (1–3 s after the injections).

There was also a statistically significant difference regarding perceived pain between the control group 2 (EMLA NaCl) and control group 3 (placebo) when administering injections of NaCl. This difference remained statistically significant up to 4 min after the injections, Fig. 3 and Table 3.

**Table 3:** Comparison between the PLACEBO and EMLA NaCl groups. VAS scores (mm) presented as mean and standard deviation (+SD) at each time-point

	PLACEBO NaCl	EMLA NaCl	
Time-points	Mean (+SD)	Mean (+SD)	p-value
1–3 s	34.0 (21,7)	6.0 (8,5)	0.000
30 s	18.6 (19,0)	6.0 (8,9)	0.000
1 min	14.0 (16,7)	2.8 (4,6)	0.000
2 min	10.6 (15,8)	2.4 (4,1)	0.008
3 min	6.17 (8,3)	1.8 (3,3)	0.005
4 min	3.8 (5,0)	1.3 (2,1)	0.028
5 min	2.0 (2,5)	1.3 (2,1)	0.116
10 min	1.3 (1,8)	1.8 (3,4)	0.916
15 min	1.4 (1,8)	2.5 (7,3)	0.236

*EMLA* Patch with local anesthesia, *NaCl* Isotonic saline (0.9%)

In total 49% (*n* = 58) of the participants did not report any sensations in the injection area 15 min after the injections were given. The remaining 51% (*n* = 61) reported one or several sensations. Overall the most common reported sensations were *sore* and *prickling*. *Sore*, *numb* and *felt warm* were more frequently reported in the SWI groups compared to NaCl groups. Moreover, the absence of sensations was more pronounced in the NaCl groups compared to the SWI groups, Table 4.

**Table 4:** Sensations reported 15 min after injections were given. In total 119 women responded to this question. Some women have reported more than one sensation

Sensation	PLACEBO and SWI	EMLA and SWI	PLACEBO and NaCl	EMLA and NaCl
Sore	9	5		1
Prickling	7	2	5	1
Stinging	6	3	4	2
Felt warm	4	4	1	1
Numb	4	4	1	
Grinding ache	4	2	1	1
Swollen	2	1		
Throbbing	2			
Unpleasant	1	1	1	1
Stiff		3	1	1
Pleasant		1		
***Total***	39	26	14	8
Reported no particular sensation	9	10	17	22

*EMLA* Patch with local anesthesia, *SWI* Sterile water injections, *NaCl* isotonic saline (0.9%)

To illustrate the variation of reported sensations in the four groups, some quotes are presented below.

**EMLA SWI**

*It feels just a bit numb and somewhat warm inside, but absolutely no pain or discomfort. I felt the lower left-hand jab a bit more than the others. The whole area is generally more sensitive than my skin used to be.*

*It feels like I have bruises and some prickling and stinging, especially on the left side. The prickling and stinging come and go every few seconds.*

**EMLA NaCl**

*Warm feeling in the area.*

*Low-grade pain. Stinging in a tennis ball-sized area on the left side. Essentially no pain at all on the right side.*

**Placebo SWI**

*I don’t feel anything different at all in the area, maybe a bit warm.*

*Prickling, with varying intensity. Like being pinched or extreme numbness at the site of injection. It’s not exactly painful but more of a stinging sensation in the spots where I was jabbed.*

**Placebo NaCl**

*I don’t feel anything, either where I got the jab or in area around it.*

*Prickling every now and then, mostly on the left. Sometimes the prickling is quite painful, but in between, I don’t feel any pain at all.*

The day after the experiment, four women reported some sensations in the injection area. In the intervention group (EMLA SWI) discomforting (*n* = 1) and sore (n = 1), in control group 1 (placebo SWI) sore (n = 1) and in control group 2 (EMLA NaCl) stiff (n = 1). No sensations were reported in control group 3 (placebo NaCl). No other adverse side effects were reported.

## Discussion

The main finding in this study was that local anesthesia with EMLA® reduces the perceived pain when administering intracutaneous (also called intradermal) injections of sterile water. Immediately after the injections were given, 70% scored mild or moderate pain (EMLA SWI group) the corresponding figure for the placebo SWI group was 37%. Our findings suggest that the use of EMLA may make the injection pain of SWI more tolerable for women.

This is the first study to report the pain and other sensations related to the injections beyond the initial treatment. At 15 min post-injection the most common reported sensations were sore, numb and felt warm. These were more frequently reported in the SWI groups compared to NaCl groups. Moreover, the absence of sensations was more pronounced in the NaCl groups compared to the SWI groups. The reason for this is unknown. However, it is relevant to assume that this has to do with the fact that sterile water is salt-free which could induce an osmotic gradient in the skin as previously described [41].

Despite that SWI has been proven to be a highly effective pain relief method, without any adverse side effects [4, 7], some women are doubtful of the method. Earlier it has been reported that some women rated the injection pain as more painful than the contraction pain [10] with a self-reported VAS of 90–100 mm [14]. The reduction of perceived pain related to the injections in the present study may mean that laboring women are more likely to use this pain relief method. Furthermore, it has been reported that midwives find it counter-intuitive to cause women additional pain when using SWI to relieve childbirth pain [11]. However, it may be more likely that midwives would recommend SWI if using an EMLA patch before treatment.

As this study aimed to establish if the use of topical anesthetic creams could reduce the pain of SWI and non-pregnant women were included as participants, there remain two essential questions that this trial was not designed to address. Firstly, as the EMLA cream takes 60–90 min to achieve an effect, would this delay in providing analgesia be acceptable to women? A woman in childbirth would, not unsurprisingly, want pain relief directly after providing her informed decision about the analgesic method for her labor pain. The delay in the effect of the EMLA and similar topical anesthetics could be too long to wait for the woman. An alternative may be to apply the EMLA patches early if the woman considers SWI a treatment for back pain, as the patches can be left in place for up to 5 h. Secondly, would the reduction in injection pain negatively impact the resulting analgesia? No studies have directly explored the relationship between injection pain and the degree of resulting analgesia. Previous studies have determined that single water injection is given at the point on the back where the woman perceives her pain to be most substantial results in a significant pain reduction [12, 13]. However, the pain of the injection was not reported. One trial compared a single to four injections and found that the single injection was significantly less painful than four and resulted in a significantly reduced analgesic effect [14]. However, women were still quite satisfied with the analgesia provided by the one injection. This suggests there may be a tradeoff between a more acceptable level of pain and a reduced but still effective degree of analgesia.

It is essential, for several reasons, to develop safe and effective non-pharmacological pain relief methods offered to women during childbirth. Many women wish to avoid methods that use drugs because of known or unknown side effects for the mother and the unborn baby. It is also essential to identify techniques that provide minimal side effects and discomfort with the highest possible pain relief. SWI has been shown to provide adequate relief of back pain in labor, however, the associated injection pain acts as a deterrent to widespread use. As the study was conducted on non-pregnant women, it is not known if reducing the injection pain will impact the degree or duration of analgesia usually associated with SWI. The next step is, therefore, to evaluate if the pain relief effect for childbirth back pain remains after local anesthesia of the skin before treatment with intracutaneous sterile water injections. The method might also be an option for women in parts of the world where there is limited access to pain relief methods during childbirth. The reduction in injection pain found in this trial may increase the utility of SWI as an option for other severe pain conditions such as ureterolithiasis or other different kinds of chronic pain.

## Strength and limitations

The study had several strengths. The trial was conducted according to a registered protocol, and only one woman discontinued participation. A number of control groups were used to explore the various combinations of EMLA versus SWI and NaCl. Limitations of the study involve the inability to blind the administration of the EMLA and placebo patches and the water and NaCl injections. The participants were unaware of the group to which they were randomized. However, there is a distinct difference regarding the pain experience between the injections of sterile water versus NaCl, which could have alerted the women of group allocation.

## Conclusion

The present study suggests that the use of topical anesthetic creams at least 1 h prior to the administration of SWI does reduce the injection pain experienced. However, the time taken for the anesthetic to take effect may prove impractical for some women or require some planning earlier in labor.

## Data Availability

The datasets generated during and or analyzed during the current study are available from the corresponding author on reasonable request.

## Abbreviations

NaCl: Isotonic saline 0.9%
SWI: Sterile Water Injections
VAS: Visual Analogue Scale
